# A novel blood pressure monitoring technique by smart HUAWEI WATCH: A validation study according to the ANSI/AAMI/ISO 81060-2:2018 guidelines

**DOI:** 10.3389/fcvm.2022.923655

**Published:** 2022-10-11

**Authors:** Lili Wang, Hong Xian, Jiajun Guo, Weihao Li, Jiaqi Wang, Qing Chen, Xiaoyu Fu, Hongbao Li, Qin Chen, Wei Zhang, Yucheng Chen

**Affiliations:** ^1^Department of Cardiology, West China Hospital, Sichuan University, Chengdu, Sichuan, China; ^2^Department of Cardiology, Fuwai Hospital, Chinese Academy of Medical Sciences and Peking Union Medical College, National Center for Cardiovascular Diseases, Beijing, China; ^3^Department of Geriatrics, West China Hospital, Sichuan University, Chengdu, Sichuan, China; ^4^Huawei Device Co., Ltd., Shenzhen, China; ^5^West China Biomedical Big Data Center, West China Hospital, Sichuan University, Chengdu, Sichuan, China

**Keywords:** smart watch, blood pressure, digital health, wearable device, validation

## Abstract

**Background:**

Given the rapid innovation of wearable technology, additional physical indicators can be detected, and blood pressure (BP) has become the focus of many emerging medical-device manufacturers. This study aimed to validate the accuracy of the newly developed HUAWEI WATCH in BP monitoring, according to the American National Standards Institute/Association for the Advancement of Medical Instrumentation/International Organization for Standardization (ANSI/AAMI/ISO 81060-2:2018) guidelines.

**Materials and methods:**

The same arm sequential BP measurement was applied. One validation included four reference BP measurements taken simultaneously by two independent observers using a mercury sphygmomanometer, alternating with three test-watch measurements. Each test-watch measurement was compared against the average of the previous and subsequent reference BP readings. Two criteria were required for validation: (1) a mean BP difference of 5 mm Hg or less, with a standard deviation (SD) of 8 mm Hg or less for systolic blood pressure (SBP) and diastolic blood pressure (DBP) in the 255 pairs of measurements, and (2) an SD for the of 85 averaged BP differences within the threshold defined by the mean test-reference BP difference listed in the ANSI/AAMI/ISO 81060-2:2018 guidelines.

**Results:**

The mean age of the 85 participants was 48 ± 18 years (range: 21–85), and 53 (62.4%) were male. The mean differences between the test and reference BPs were -0.25 ± 5.62 mm Hg and -1.33 ± 6.81 mm Hg for SBP and DBP, respectively (according to Criterion 1). The mean differences between the test BPs and reference BPs were -0.25 ± 5.00 mm Hg and -1.33 ± 6.31 mm Hg for SBP and DBP, respectively, according to Criterion 2.

**Conclusion:**

Blood pressure measurement using the HUAWEI WATCH showed excellent consistency with reference BPs, and fulfilled both validation criteria of the guidelines, show its promise as a wearable device for BP self-monitoring.

## Introduction

Hypertension is one of the most important preventable causes of premature morbidity and mortality. It affects more than 1 billion persons globally, and accounts for 10 million deaths worldwide per annum (1). The accurate measurement of blood pressure (BP) is essential in the management of hypertension, which requires a standardized procedure and a validated device.

Out-of-office BP measurement is widely used and recommended by both European and American guidelines (2, 3), for the following reasons. Out-of-office BP measurements are usually lower than conventional office BP measurements, which may reduce or eliminate the “white-coat” effect. Out-of-office BP measurements provide BP data that are more reproducible, which may be helpful in detecting “masked hypertension.” Out-of-office self-monitoring BP may have a beneficial effect on medication adherence and BP control (4–6). Out-of-office BP is more closely related to hypertension-mediated organ damage (7), and it is a better predictor of cardiovascular morbidity and mortality than office measurements of BP (8). Out-of-office BP measurements are typically taken early in the morning and at bedtime, as daytime BP level is often overlooked. Although recent studies have found that daytime stress at the workplace may increase BP, the prevalence of hypertension has been found to be high at the workplace, while awareness and control of it is poor (9, 10). Therefore, a portable BP device that can monitor BP anywhere and anytime may help to improve the condition.

Wearable devices are widespread, and an increasing number of adults are using smartwatches or wrist-worn fitness bands. Many physical indicators, such as heart rate, heart rhythm, electrocardiogram, oxygen saturation, and sleep can be detected using wearable devices (11), but BP cannot be accurately measured using a wearable device. Watch-based BP measurement equipment can be a great convenience to the user. The newly developed HUAWEI WATCH (HUAWEI Technologies Co. Ltd., Shenzhen, China) is equipped with a BP measuring function, and to our knowledge, it is the first smartwatch equipped with a BP measurement function. Therefore, the present study aimed to validate the accuracy of the HUAWEI WATCH in BP monitoring according to the guidelines of the American National Standards Institute/Association for the Advancement of Medical Instrumentation/International Organization for Standardization (ANSI/AAMI/ISO 81060-2:2018) (12).

## Materials and methods

### Study subjects

This study was conducted and reported following the Strengthening the Reporting of Observational Studies in Epidemiology (STROBE) statement. This study was approved by the ethics committee of our institution, and all of the participants gave their informed consent to participate. The clinical trial registration number is ChiCTR2000040197. Participants were recruited as volunteers, and the inclusion criterion was age ≥18 years. The exclusion criteria were: (i) arrhythmia, inaudible phase V Korotkoff sounds to determine the DBP, (ii) inability to cooperate with blood pressure measurements, and (iii) a wrist circumference of <13.0 cm or >20.0 cm.

### Features of the device

**FIGURE 1:**
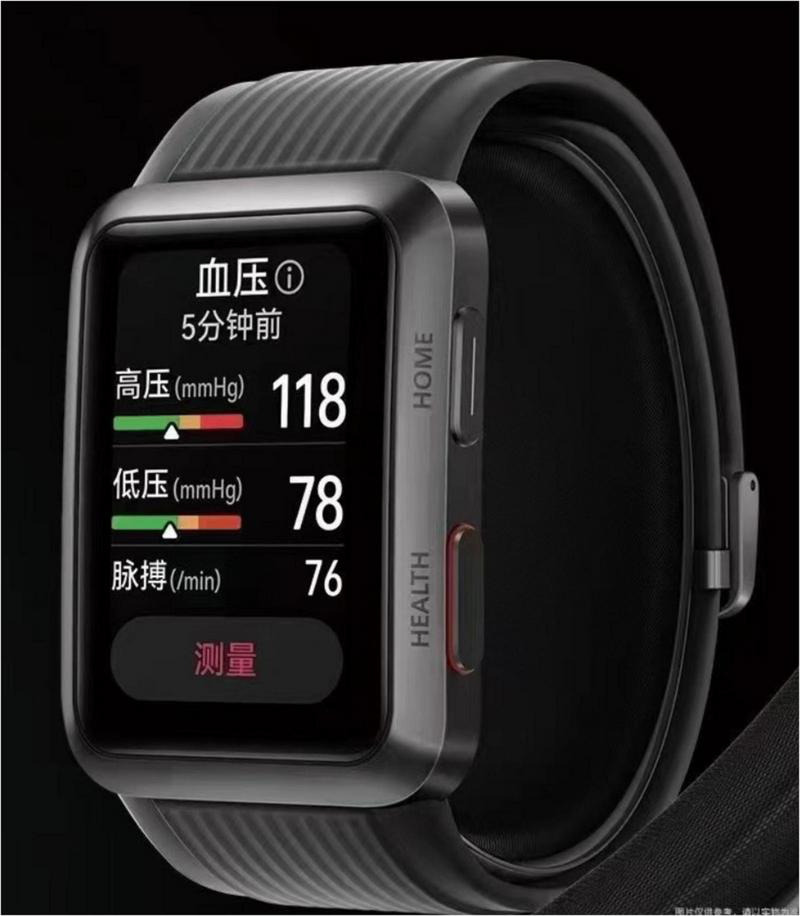
Illustration of the HUAWEI WATCH.

The newly developed HUAWEI WATCH is equipped with a BP measuring function (Figure 1). The BP measurement of the WATCH is based on oscillometry, which involves using a micro-pump and a detachable cuff. Two cuffs of different sizes are provided to accommodate different wrist circumferences.

The measurement range of the HUAWEI WATCH is 60–230 mm Hg for systolic BP (SBP) and 40–160 mm Hg for diastolic BP (DBP). It analyzes the pulse wave detected during inflation using an algorithm for determining the SBP and DBP; the algorithm is proprietary and cannot be disclosed at this time.

### Blood pressure validation

**FIGURE 2:**
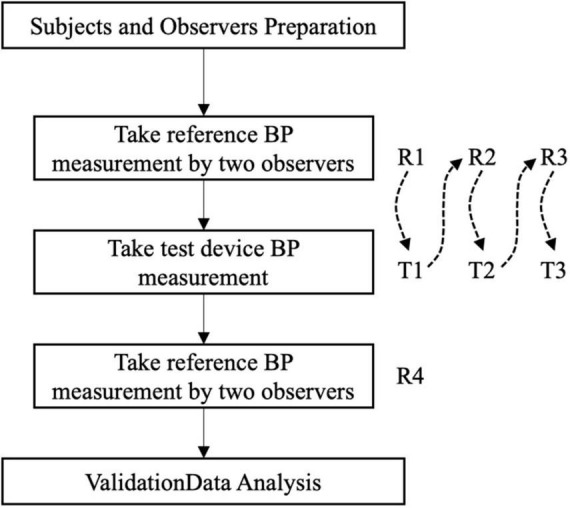
The same arm sequential BP measurement process.

The same arm sequential BP measurement was applied in accordance with the ANSI/AAMI/ISO 81060-2:2018 guidelines. The measurements were taken in a quiet room, after a 5-min rest period. During the process, the participants remained quiet with their legs uncrossed in a sitting position. One validation included four reference BP measurements (R1-R4), alternating with three test-watch measurements (R1-T1-R2-T2-R3-T3-R4), as shown in Figure 2.

The reference BP measurements were taken simultaneously by two independent observers using a Y-tube and a calibrated mercury sphygmomanometer. Participants’ SBP was determined based on phase I Korotkoff sounds heard by the observer, and DBP was determined based on phase V disappearance of the Korotkoff sounds. A third observer served as a supervisor who checked the BP readings of the two observers. Any pair of SBP or DBP observations with a difference greater than 4 mm Hg was excluded, and another group of measurements was performed. Measurements of BP using the mercury sphygmomanometer were recorded as the average value of the BPs measured by the two observers. The reference BPs was recorded as the average value of the previous and subsequent BP readings by the mercury sphygmomanometer. If the previous and subsequent reference SBP readings differed by more than 12 mm Hg, or the DBP readings differed by more than 8 mm Hg, all data from the participants were excluded as cases of “Reference BP variations.” Participants’ BP was measured with their left wrist positioned at the level of the heart, and the time between each set of BP measurements was at least 60 s.

### Statistical analysis

Normally distributed continuous variables are expressed as mean ± standard deviation (SD), and categorical variables are expressed as number and percentage. Data were analyzed in accordance with Criteria 1 and 2 of the ANSI/AAMI/ISO 81060-2:2018 guidelines. For Criterion 1, each test BP reading minus the reference BP reading by a mercury sphygmomanometer were calculated, for a total of three differences for each participant. The mean and SD of the difference was calculated to fulfill the Criterion 1 requirement for a mean BP difference of 5 mm Hg or less for 255 pairs of measurements, and an SD of 8 mm Hg or less for SBP and DBP. For Criterion 2, a difference was defined as the mean of the three test SBPs or DBPs, as measured by the HUAWEI WATCH minus the mean values of the three reference SBPs or DBPs. A total of 85 pairs of BP differences were calculated to fulfill Criterion 2; the SDs of the 85 pairs of BP differences were required to be within the threshold defined by the mean test-reference BP difference listed in the ANSI/AAMI/ISO 81060-2:2018 (see Table 1 for SBP and DBP). The data were analyzed using SPSS 26.0 (IBM Corp., Armonk, NY, USA) on software, version 3.8.8 (G. van Rossum). Data analyses were conducted in February 2022.

**TABLE 1:** Characteristics of the study participants.

Age, years	48 ± 18
Men: women, n	53:32
Height, cm	169.9 ± 8.0 (147.0–183.0)
Weight, kg	62.8 ± 13.7 (36.0–96.0)
Wrist circumference, mm (range)	162.13 ± 15.64 (128.00–197.00)
**Distribution of SBPs**	
≥160 mm Hg, n (%)	7 (8.3%)
140–160 mm Hg, n (%)	11 (12.9%)
100–140 mm Hg, n (%)	47 (55.3%)
≤100 mm Hg, n (%)	20 (23.5%)
**Distribution of DBPs**	
≥100 mm Hg, n (%)	8 (9.4%)
85–100 mm Hg, n (%)	9 (10.6%)
60–85 mm Hg, n (%)	60 (70.6%)
≤60 mmHg, n (%)	8 (9.4%)

SBP, systolic blood pressure; DBP, diastolic blood pressure.

## Results

In total, 107 participants were screened, 22 were excluded, and 85 sets of valid measurements were analyzed to comprise the final participant group. The participants’ mean age was 48 ± 18 years (range: 21–85 years), 53 (62.3%) were men, 32 (37.7%) were women, and the gender distribution fulfilled the guideline’s 30% criterion. Participants’ mean height was 169.9 ± 8.0 cm (range: 147.0–183.0 cm), their mean weight was 62.8 ± 13.7 kg (range: 36.0–96.0 kg), and their mean wrist circumference was 162.13 ± 15.64 mm (range: 130.00–197.00 mm). The characteristics of the 85 participants are summarized in Table 1. Distribution of the reference BPs fulfilled the criterion stated in the guidelines, with high (≥160 mm Hg), medium (≥140 mm Hg), and low (≤100 mm Hg) percentages of 8.3% (meeting the 5% criterion), 21.1% (20% criterion), and 23.5% (5% criterion), respectively, for the reference SBPs. The high (≥100 mm Hg), medium (≥85 mm Hg), and low (≤60 mm Hg) percentages were respectively, 9.4% (meeting the 5% criterion), 20.0% (20% criterion), and 9.4% (5% criterion), respectively, for reference DBPs, as shown in Table 1.

**FIGURE 3:**
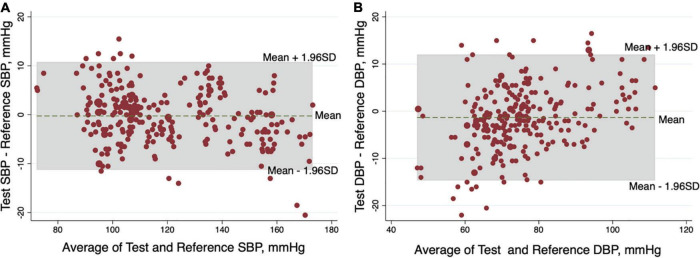
Bland-Altman plots of the differences between the test blood pressures (BPs) by HUAWEI WATCH and the reference BPs by mercury sphygmomanometer for the systolic blood pressure (SBP) **(A)** and diastolic blood pressure (DBP) **(B)**.

The mean differences between the test-watch and reference BPs were −0.25 ± 5.62 mm Hg for SBP and −1.33 ± 6.81 mm Hg for DBP, in accordance with Criterion 1. The results are presented in Table 2. The Bland–Altman analysis showed a bias of −0.25 with limits of agreement ranging from −11.27 to 10.54 mm Hg for SBP (Figure 3A) and a bias of −1.33 with limits of agreement from −14.56 to 12.02 for DBP (Figure 3B). The mean differences between the test-watch and reference BPs were −0.25 ± 5.00 mm Hg for SBP and −1.33 ± 6.31 mm Hg for DBP in accordance with Criterion 2 (Table 2). These results fulfilled the ANSI/AAMI/ISO 81060−2:2018 validation criteria of ≤5 ± ≤8.0 mm Hg for Criterion 1, and SDs of <6.95 mm Hg for SBP and <6.82 mm Hg for the DBP for Criterion 2.

**TABLE 2:** Validation results in accordance with Criterion 1 and Criterion 2 of the guidelines.

	SBP	DBP
Criterion 1	-0.25 ± 5.62	-1.33 ± 6.81
Criterion 2	-0.25 ± 5.00	-1.33 ± 6.31

SBP, systolic blood pressure; DBP, diastolic blood pressure.

## Discussion

In the present study, we validated the performance of the HUAWEI WATCH’s monitoring of BP in accordance with the ANSI/AAMI/ISO 81060-2:2018 guidelines. The results showed that the HUAWEI WATCH fulfilled Criteria 1 and 2 of the guidelines, indicating that it could be a reliable and convenient device in the daily self-monitoring of BP.

Usually, BP measurements (or self-monitored BP measurements) are taken in the home, typically in the morning and at bedtime, thereby overlooking daytime BP levels. Given the research findings that BP levels increase throughout the day, and that daily variability of BP is a predictor of future cardiovascular events (13), the awareness of daytime BP measurements has increased through research findings on daytime BP, especially at the workplace. A recent clinical trial found that the prevalence of hypertension was high among the working population in China, but the rates of awareness, treatment, and control of BP were unacceptably low, indicating there is substantial room for improvement in the diagnosis and treatment of hypertension among employees at the workplace (9). Another study found that workplace-based interventions, which improved hypertension control, appeared to be more effective than usual care (10). However, a previous study with similar findings demonstrated that BP measured in the workplace was more closely related to left ventricular mass index than was BP measured in a clinic (14). A sphygmomanometer is not a convenient measurement tool in the workplace; it is a heavy and cumbersome instrument. However, a portable and compact device, such as a watch-type of wearable BP monitor is ideal in workplace settings.

The Omron HEM-6410T-ZM and Omron HEM-6410T-ZL were the first wristwatch types of wearable BP monitors. The mean differences between the test and reference SBPs were −0.9 and −1.1 mm Hg for the two devices, respectively; the mean differences for the DBPs were 2.4 and 0.3 mm Hg, and both devices fulfilled the validation criteria of the ANSI/AAMI/ISO81060-2:2018 guidelines (15). The mean differences between the test and reference BPs were −0.25 and −1.33 mm Hg for the SBP and DBP, respectively, for the HUAWEI WATCH, which was smaller than the Omron watch-type wearable BP monitor. In addition, the range of the wrist-circumferences accommodated by the Omron watch-type wearable BP monitors were very narrow (16–21.5 cm). In our study, the wrist circumference of many of the participants was below 16 cm; thus, the HUAWEI WATCH, which has a wrist circumference of 13–20 cm, was suitable for more participants. The HUAWEI WATCH enables consumers to measure their BP frequently, throughout their activities of daily living, and most importantly, the HUAWEI WATCH is a smartwatch-based BP monitor, equipped with other functions, and therefore, more consistent with the needs of today’s society.

### Limitations

One study limitation is that the HUAWEI WATCH was validated using participants’ left wrist at heart level; hence, further validation is needed in future studies.

## Conclusion

In conclusion, BP measurements using the HUAWEI WATCH were consistent with the reference BPs and fulfilled both of the guidelines’ validation criteria, thereby showing its promise as a wearable device for BP self-monitoring.

## Data availability statement

The raw data supporting the conclusions of this article will be made available by the authors, without undue reservation.

## Ethics statement

The studies involving human participants were reviewed and approved by West China Hospital, Sichuan University. The patients/participants provided their written informed consent to participate in this study.

## Author contributions

YC: concept and design. LW, JG, WL, JW, HX, QingC, QinC, HL, and XF: acquisition, analysis, and interpretation of the data. LW, HX, JG, WL, and JW: drafting of the manuscript. YC and WZ: critical revision of the manuscript. LW, HX, JG, and WL: statistical analysis. LW, JG, WL, QingC, QinC, HL, and XF: administrative, technical, or material support. WZ and YC: supervision. All authors contributed to the article and approved the submitted version.

## References

[1] Forouzanfar MH Liu P Roth GA Ng M Biryukov S Marczak L Global burden of hypertension and systolic blood pressure of at least 110 to 115 mm Hg, 1990-2015. *JAMA.* (2017) 317:165–82. 28097354 10.1001/jama.2016.19043

[2] Williams B Mancia G Spiering W Agabiti Rosei E Azizi M Burnier M 2018 ESC/ESH guidelines for the management of arterial hypertension. *Eur Heart J.* (2018) 39:3021–104. 30165516 10.1093/eurheartj/ehy339

[3] Whelton PK Carey RM Aronow WS Casey DE Jr. Collins KJ Dennison Himmelfarb C 2017 ACC/AHA/AAPA/ABC/ACPM/AGS/APhA/ASH/ASPC/NMA/PCNA guideline for the prevention, detection, evaluation, and management of high blood pressure in adults: executive summary: a report of the American college of cardiology/American heart association task force on clinical practice guidelines. *J Am Coll Cardiol.* (2018) 71:2199–269. 10.1161/HYP.0000000000000075 29146533

[4] McManus RJ Mant J Haque MS Bray EP Bryan S Greenfield SM Effect of self-monitoring and medication self-titration on systolic blood pressure in hypertensive patients at high risk of cardiovascular disease: the TASMIN-SR randomized clinical trial. *JAMA.* (2014) 312:799–808. 10.1001/jama.2014.10057 25157723

[5] McManus RJ Mant J Bray EP Holder R Jones MI Greenfield S Telemonitoring and self-management in the control of hypertension (TASMINH2): a randomised controlled trial. *Lancet.* (2010) 376:163–72. 10.1016/S0140-6736(10)60964-6 20619448

[6] Tucker KL Sheppard JP Stevens R Bosworth HB Bove A Bray EP Self-monitoring of blood pressure in hypertension: a systematic review and individual patient data meta-analysis. *PLoS Med.* (2017) 14:e1002389. 10.1371/journal.pmed.1002389 28926573 PMC5604965

[7] Bliziotis IA Destounis A Stergiou GS. Home versus ambulatory and office blood pressure in predicting target organ damage in hypertension: a systematic review and meta-analysis. *J Hypertens.* (2012) 30:1289–99. 10.1097/HJH.0b013e3283531eaf 22499289

[8] Ward AM Takahashi O Stevens R Heneghan C. Home measurement of blood pressure and cardiovascular disease: systematic review and meta-analysis of prospective studies. *J Hypertens.* (2012) 30:449–56. 10.1097/HJH.0b013e32834e4aed 22241136

[9] Shen Y Wang X Wang Z Zhang L Chen Z Zhu M Prevalence, awareness, treatment, and control of hypertension among Chinese working population: results of a workplace-based study. *J Am Soc Hypertens.* (2018) 12:311–22.e2. 10.1016/j.jash.2018.01.013 29483001

[10] Wang Z Wang X Shen Y Li S Chen Z Zheng C Effect of a workplace-based multicomponent intervention on hypertension control: a randomized clinical trial. *JAMA Cardiol.* (2020) 5:567–75. 10.1001/jamacardio.2019.6161 32129791 PMC7057176

[11] Sim I. Mobile devices and health. *N Engl J Med.* (2019) 381:956–68. 10.1056/NEJMra1806949 31483966

[12] International Organization for Standardization. *ISO 81060-2:2018. Non-Invasive Sphygmomanometers-Part 2: Clinical Investigation of Intermittent Automated Measurement Type.* Geneva: ISO (2018).

[13] Chowdhury EK Owen A Krum H Wing LM Nelson MR Reid CM Systolic blood pressure variability is an important predictor of cardiovascular outcomes in elderly hypertensive patients. *J Hypertens.* (2014) 32:525–33. 10.1097/HJH.0000000000000028 24481213

[14] Devereux RB Pickering TG Harshfield GA Kleinert HD Denby L Clark L Left ventricular hypertrophy in patients with hypertension: importance of blood pressure response to regularly recurring stress. *Circulation.* (1983) 68:470–6. 10.1161/01.CIR.68.3.470 6223721

[15] Kuwabara MHK Hishiki Y Kario K. Validation of two watch-type wearable blood pressure monitors according to the ANSI/AAMI/ISO81060-2:2013 guidelines: Omron HEM-6410T-ZM and HEM-6410T-ZL. *J Clin Hypertens (Greenwich).* (2019) 21:853–8. 10.1111/jch.13499 30803128 PMC8030427

